# Characterizing the Community Structure of Complex Networks

**DOI:** 10.1371/journal.pone.0011976

**Published:** 2010-08-12

**Authors:** Andrea Lancichinetti, Mikko Kivelä, Jari Saramäki, Santo Fortunato

**Affiliations:** 1 Complex Networks and Systems Lagrange Laboratory, Institute for Scientific Interchange (ISI), Torino, Italy; 2 Department of Biomedical Engineering and Computational Science, Aalto University, Espoo, Finland; Indiana University, United States of America

## Abstract

**Background:**

Community structure is one of the key properties of complex networks and plays a crucial role in their topology and function. While an impressive amount of work has been done on the issue of community detection, very little attention has been so far devoted to the investigation of communities in real networks.

**Methodology/Principal Findings:**

We present a systematic empirical analysis of the statistical properties of communities in large information, communication, technological, biological, and social networks. We find that the mesoscopic organization of networks of the same category is remarkably similar. This is reflected in several characteristics of community structure, which can be used as “fingerprints” of specific network categories. While community size distributions are always broad, certain categories of networks consist mainly of tree-like communities, while others have denser modules. Average path lengths within communities initially grow logarithmically with community size, but the growth saturates or slows down for communities larger than a characteristic size. This behaviour is related to the presence of hubs within communities, whose roles differ across categories. Also the community embeddedness of nodes, measured in terms of the fraction of links within their communities, has a characteristic distribution for each category.

**Conclusions/Significance:**

Our findings, verified by the use of two fundamentally different community detection methods, allow for a classification of real networks and pave the way to a realistic modelling of networks' evolution.

## Introduction

The modern science of complex systems has experienced a significant advance after the discovery that the graph representation of such systems, despite its simplicity, reveals a set of crucial features that suffice to disclose their general structural properties, function and evolution mechanisms [1]–[8]. Representing a complex system as a graph means turning the elementary units of the system into nodes, while links between nodes indicate their mutual interactions or relations. Many complex networks are characterized by a broad distribution of the number of neighbors of a node, *i.e.* its degree. This is responsible of peculiar properties such as high robustness against random failures [9] and the absence of a threshold for the spreading of epidemics [10].

Another important feature of complex networks is represented by their mesoscopic structure, characterized by the presence of groups of nodes, called communities or modules, with a high density of links between nodes of the same group and a comparatively low density of links between nodes of different groups [11]–[14]. This compartmental organization of networks is very common in systems of diverse origin. It was remarked already in the 1960's that a hierarchical modular structure is necessary for the robustness and stability of complex systems, and gives them an evolutionary advantage [15].

Exploring network communities is important for three main reasons: 1) to reveal network organization at a coarse level, which may help to formulate realistic mechanisms for its genesis and evolution; 2) to better understand dynamic processes taking place on the network (e.g., spreading processes of epidemics and innovation), which may be considerably affected by the modular structure of the graph; 3) to uncover relationships between the nodes which are not apparent by inspecting the graph as a whole and which can typically be attributed to the function of the system.

Therefore it is not surprising that the last years have witnessed an explosion of research on community structure in graphs. The main problem, of course, is how to detect communities in the first place, and this is the essential issue tackled by most papers on the topic which have appeared in the literature. A huge number of methods and techniques have been designed, but the scientific community has not yet agreed on which methods are most reliable and when a method should or should not be adopted. This is due to the fact that the concept of community is ill-defined. Since the focus has been on method development, very little has been done so far to address a fundamental question of this endeavor: *what do communities in real networks look like?* This is what we will try to assess in this paper.

Previous investigations have shown that across a wide range of networks, the distribution of community sizes is broad, with many small communities coexisting with some much larger ones [12], [16]–[19]. The tail of the distribution can be often quite well fitted by a power law. Leskovec et al. [20] have carried out a thorough investigation of the quality of communities in real networks, measured by the conductance score [21]. They found that the lowest conductance, indicating well-defined modules, is attained for communities of a characteristic size of 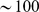 nodes, whereas much larger communities are more “mixed” with the rest of the network. For this reason they suggest that the mesoscopic organization of networks may have a core-periphery structure, where the periphery consists of small well-defined communities and the core comprises larger modules, which are more densely connected to each other and therefore harder to detect. Guimerá and Amaral have proposed a classification of the nodes based on their roles within communities [22].

However, the fundamental properties of communities in real networks are still mostly unknown. Uncovering such properties is the main goal of this paper. For this purpose, we have performed an extensive statistical analysis of the community structure of many real networks from nature, society and technology. The main conclusion is that communities are characterized by distinctive features, which are common for networks of the same class but differ from one class to another. Remarkably, such characterization is independent of the specific method adopted to find the communities.

## Methods

As our target is to study the statistical features of communities, we need to employ data sets on large networks containing high numbers of communities of varying size. Our data sets contain 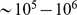 nodes, with exception for protein interaction networks (PINs), where the largest available data sets are of the order of 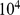 nodes.

Table 1 lists the network datasets we have used, along with some basic statistics. Most of them have been downloaded from the Stanford Large Network Dataset Collection (http://snap.stanford.edu/data/). Some networks are originally directed (e.g., the Web graph), but we have treated them as undirected. Further details on all networks can be found in the Appendix S1.

**Table 1:** List of the network datasets used for our analysis.

Network statistics					
Category	name	# nodes	# links	average degree	max degree
Communication	wikitalk	2,394,385	4,659,560	3.89	100,029
	email	265,214	364,481	2.75	7,636
Internet	caida	26,475	53,381	4.03	2,628
	dimes	26,211	76,261	5.82	3,988
Information	Web google	875,713	4,322,050	9.87	6,332
	arxiv	27,770	352,285	25.37	2,468
	amazon	410,236	2,439,440	11.89	2,760
	Web BS	685,230	6,649,470	19.41	84,230
Biological	dmela	7,498	22,678	6.05	178
	yeast	1,870	2,203	2.36	56
	human	4,998	21,747	8.70	282
Social	live j	4,846,609	42,851,211	17.68	20,333
	epinions	75,879	405,740	10.69	3,044
	last fm	2,647,364	11,245,707	8.49	13,431
	slashdot	773,60	469,180	12.13	2,539

For each network we specify the number of nodes and links, the average and maximum degree.

Overall, we have considered five categories of networks:

- **Communication networks.** This class comprises the email network of a large European research institution, and a set of relationships between Wikipedia users communicating via their discussion pages. Note that in both cases, communication is not necessarily personal but involves, e.g., mass emails, and thus these networks cannot be considered as social networks.
- **Internet.** Here we have two maps of the Internet at the Autonomous Systems (AS) level (i.e. nodes are groups of routers administered by a single entity), produced by the two main projects exploring the topology of the Internet: CAIDA (http://www.caida.org/) and DIMES (http://www.netdimes.org/).
- **Information networks.** This class includes a citation network of online preprints in www.arxiv.org, a co-purchasing network of items sold by www.amazon.com and two samples of the Web graph, one representing the domains berkeley.edu and stanford.edu (Web-BS), the other was released by Google (Web-G).
- **Biological networks.** This class contains the sets of interactions between proteins of three organisms: fruit fly (*Drosophila melanogaster*), yeast (*Saccharomyces cerevisiae*) and man (*Homo sapiens*).
- **Social networks.** Here we considered four datasets: a network of friendship relationships between users of the on-line community *LiveJournal* (www.livejournal.com); the set of trust relationships between users of the consumer review site epinions.com; the friendship network of users of slashdot.org; the friedship network of users of www.last.fm.

The problem of choosing a method for detecting communities is a very delicate one. First, very efficient algorithms are needed, because the networks we study are large. This requirement rules out the majority of existing methods. Second, as discussed above, there is no common agreement on an all-purpose community detection method. This is because of the absence of a shared definition of community, which is justified by the nature of the problem itself. Consequently, there is also arbitrariness in defining reliable testing procedures for the algorithms. Nevertheless, there is a wide consensus on the definition of community originally introduced in a paper by Condon and Karp [23]. The idea is that a network has communities if the probability that two nodes of the same community are connected exceeds the probability that nodes of different communities are connected. This concept of community has been implemented to create classes of benchmark graphs with communities, such as those introduced by Girvan and Newman [11] and the graphs recently designed by Lancichinetti et al. [24], which integrate the benchmark by Girvan and Newman with realistic distributions of degree and community size (LFR benchmark). Recent work indicates that some algorithms perform very well on the LFR benchmark [25]. In particular, the Infomap method introduced by Rosvall and Bergstrom [26] has an outstanding performance, and it is also fast and thus suitable for large networks. However, as every community detection method has its own “flavor” and preference towards labeling certain types of structure as communities, relying on a single method is not enough if general conclusions on community structure are to be presented. Therefore we have cross-checked the results obtained by Infomap with those produced by a very different algorithm, the Label Propagation Method (LPM) proposed by Leung et al. [27]. The latter has proven to be reliable on the LFR benchmark and is also fast enough to handle the largest systems of our collection. Detailed descriptions of Infomap and the LPM are given in Appendix S1. Here we just point out the profound differences between the two techniques. Infomap is a global optimization method, which aims to optimize a quality function expressing the code length of an infinitely long random walk taking place on the graph. The LPM is a local method instead, where nodes are attributed to the same community where most of their neighbors are. The partitions obtained by both methods for the same network are in general different. However, the general statistical features of community structure do not appear to depend much on the details of partitions. In the following, only Infomap results will be presented; for LPM, see Appendix S1.

## Results

**Figure 1:**
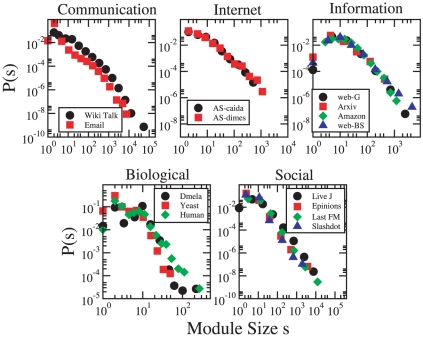
Distribution of community sizes. **All distributions are broad, and similar for systems in the same category.** Data points are averages within logarithmic bins of the module size 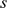.

We begin the analysis by briefly discussing the distribution of community sizes (Fig. 1). We see that, as expected, for each system there is a wide range of community sizes, spanning several orders of magnitude for the largest systems. This is in agreement with earlier studies [12], [16]–[19]. The overall shapes of the distributions are similar across systems of the same class. Distributions for biological networks show the largest differences, which, however, is likely to result from noise as the networks are smaller. For biological networks, analysis performed with the LPM shows slightly different, well overlapping distributions (see Appendix S1).

Next, we turn to the topology of the communities, and study the link density of communities and its dependence on community size. The link density of a subgraph is defined as the fraction of existing links to possible links, 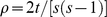, where 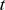 is the number of its internal links and 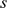 its size measured in nodes. Here, we use the scaled link density 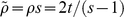, which also approximately amounts to the average internal degree of nodes in the community. We have chosen this measure since it clearly points out the nature of subgraphs. For trees, there are always 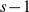 links, and hence 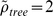. On the other hand, for full cliques 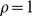 and hence 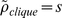.

**Figure 2:**
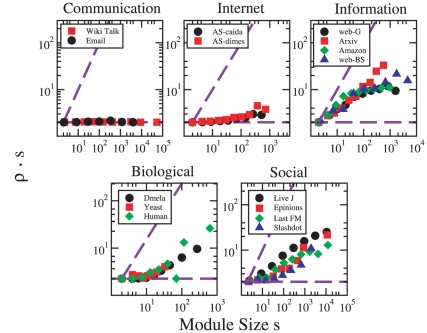
Scaled link density of communities as a function of the community size. Communication and Internet networks consist of essentially tree-like communities, while communities of social and information networks are much denser. Small modules in biological networks are often tree-like, while larger modules are denser. Data points are averages within logarithmic bins of the module size 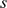.

**Figure 3:**
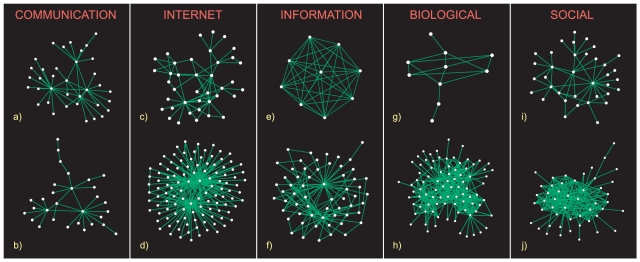
Visualized examples of communities in networks of different classes. Communication networks (a: email, b: Wiki Talk) contain very sparse communities with star-like hubs. These hubs give rise to very low shortest path lengths within communities (see Fig. 2). Star-like hubs are also present in Internet communities (c: DIMES, d: CAIDA), which are relatively sparse as well. The CAIDA community displays a “merged-star” structure fairly typical for these networks (see Appendix S1). On the contrary, information networks contain dense communities up to large cliques (e: Amazon, f: Web-BS). In biological networks, the larger the community, the less tree-like it is (g: D. melanogaster, h: H. sapiens). Finally, communities in social networks appear on average fairly homogeneous (i: Slashdot, j: Epinions).

Fig. 2 displays the average scaled link densities 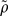 as function of community size for different networks. The dashed lines indicate the limiting cases (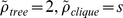). We see that the link densities in the communication and Internet networks are very close to the lower limit, which means that their communities are tree-like and contain only few or no loops. In communication networks, the scaled link density does not depend on community size, whereas in Internet graphs large communities appear somewhat denser. Networks in these two classes are the sparsest in our collection, as their very small average degree indicates that they are overall not much denser than trees (see Table 1). It should be noted that in general, the intuitive view on communities is that they are “dense” compared to the rest of the network. However, as the methods applied here yield partitions, the communities of a tree-like network are also necessarily tree-like. Contrary to the above, the much denser information networks reveal a different picture, where communities are fairly dense objects, with the scaled density increasing with 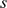. Especially in the Amazon network, communities with 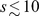 are almost cliques. Social networks show yet another pattern: the scaled density of the modules grows quite regularly with the size 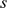, approximately as a power law. Communities in social networks are mostly far from the two limiting cases: they are denser than trees, but much sparser than cliques, with the exception of small communities which appear more tree-like. Finally, the biological networks are characterized by two regimes: for 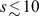, communities are very tree-like; for larger values of 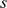 the scaled density increases with 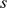. In Fig. 3 the characteristic communities of each network class are illustrated.

**Figure 4:**
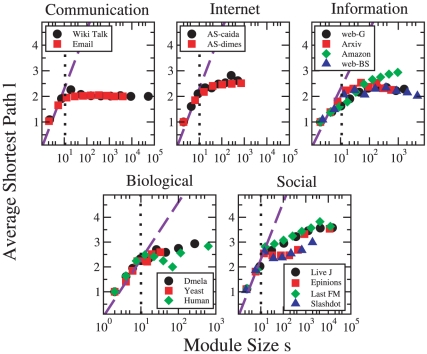
Average shortest path lengths 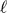 within communities as a function of community size 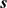. After an initial logarithmic “small-world” regime (dashed diagonal line), the average shortest path grows much slower or saturates for communities with 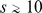 nodes (dotted vertical line). Data points are averages within logarithmic bins of module size 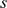.

The compactness of communities can be measured using the average shortest path length 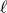 within each community. Fig. 4 displays the average values of 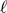 as function of community size 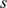. For all networks, the average shortest path lengths 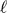 are very small, 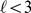 with the exception of social networks. Interestingly, all plots reveal the same basic pattern, independently of the network class. For very small communities, 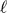 grows approximately as the logarithm of the community size (indicated by the dashed line), which is the “small-world” property typically observed in complex networks [28]. We call these modules *microcommunities*. For sizes 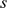 of the order of 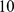, however, the increase of 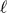 suddenly becomes less pronounced, and several curves reach a plateau. Modules with 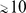 nodes are *macrocommunities*. The stabilization of the average shortest path length in macrocommunities can be attributed to the presence of nodes with high degree, *i.e.* hubs, which make geodesic paths on average short. We remark that, since most of our systems have broad degree distributions, shortest path lengths are very short [29], but the sharp transition we observe is unexpected and appears as an entirely novel feature.

For communication networks, there is a plateau with 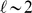 for 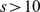. As these communities are tree-like, this indicates that they have a star-like structure where most nodes are connected to a central hub only and thus their distance equals two. For the Internet networks, the joint presence of low density and low distances also means that hubs dominate the structure – here, “merged-star” structures consisting of two or more hubs sharing many of their neighbors were observed (see Fig. 3d). This structure guarantees an efficient communication between the systems' units. On the contrary, information, social, and biological networks have a higher density and hence their short path lengths are due to both the density and the presence of hubs. Hubs play the least dominant role in social networks, as the average shortest path lengths keep slowly increasing also for large 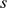.

**Figure 5:**
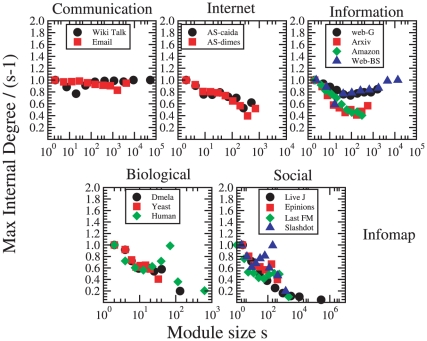
The maximal observed internal degree of nodes as a function of the community size 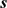. This quantity equals one if any node is linked to all other nodes of its community, and thus quantifies the dominance of hubs within communities.

The above picture is further corroborated by Fig. 5, which displays the ratio between the maximal observed community-internal degree of nodes 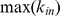 and 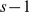 as a function of the community size 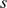. This ratio equals unity if any node is connected to all other nodes in its community, and thus it quantifies the dominance of the biggest hubs within communities. For communication networks, 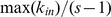 is close to unity even for large 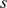, in accordance with the above observations on star-like communities. For Internet, this quantity somewhat decreases with 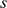, as communities may contain multiple hubs which do not connect to all other nodes. In information networks, there are some differences. In the Web graphs, the largest communities contain nodes connecting (almost) the entire community. As the edge density in these communities is high, there may be several such nodes – in a clique, all nodes have degree 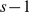. For biological and social networks, there is a decreasing trend. Especially in social networks, there are few or no dominant hubs in large communities. We remark that the agreement between the curves of Fig. 5 is more qualitative than quantitative (especially for social and biological networks), at variance with other signatures. This is because the plots refer to the properties of a very restricted class of “extremal” nodes, i.e. of the community hubs. So, on the one hand, the noise of the curves is larger. On the other hand, community detection methods have different ways to treat hubs: while methods generally tend to put them “within” communities, others (like Infomap) occasionally place them “between” communities.

**Figure 6:**
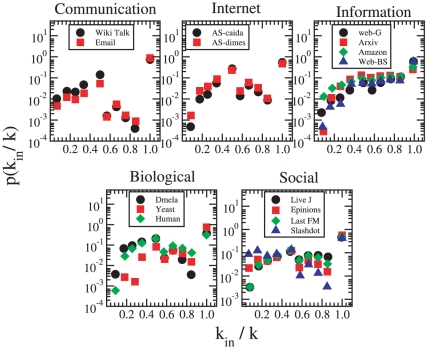
Probability distribution for isa 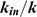, the fraction of neighbors of a node belonging to its own community. Networks in the same class display similar behavior.

Let us next take a closer look at the relationship between individual nodes and community structure. Here, the most natural property to investigate is the internal degree 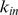, indicating the number of neighbors of a node in its community. We measure the *embeddedness* of a node in its community with the ratio 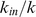, characterizing the extent to which the node's neighborhood belongs to the same community as the node itself. The probability distribution of the embeddedness ratio of all nodes in their respective networks is displayed in Fig. 6. One would straighforwardly assume that on average, the embeddedness of nodes would be fairly large, and a substantial fraction of their neighbors should reside inside their respective communities. However, Fig. 6 shows a more intricate pattern, where smaller values of 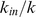 are not at all rare. All of our networks are characterized by a substantial fraction of nodes which are entirely internal to their communities, *i.e.* have no links to outside their community and thus 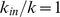. These correspond to the rightmost data points in each plot, and such nodes typically amount to over 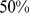 of all nodes. These nodes have mostly a low degree (such as the degree-one nodes connected to hubs in communication networks). Networks in the same class follow essentially a very similar pattern. Communication networks and the Internet have very similar-looking profiles, where the distribution has a peak around 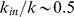. Information networks, instead, have a rather different profile, with an initial smooth increase reaching a plateau at about 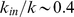. The biological networks, despite the inevitable noise, also show a consistent picture across datasets. They somewhat resemble the communication and Internet networks, with an initial rise until 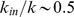, followed by a slow descent for larger values. Social networks have a rather flat distribution over the whole range, with little variations from one system to another. This means that there are many nodes with most of their neighbors outside their own community. Most community detection techniques, including the ones we have adopted, tend to assign each node to the community which contains the largest fraction of its neighbors. This implies that if a node has only a few neighbors within its own community, it will have even fewer neighbors within other individual communities. Such nodes act as “intermediates” between many different modules, and are shared between many communities rather than belonging to a single community only. Hence it would be more correct to assign them to more than one community. Overlapping communities are known to be very common in social networks, and dedicated techniques for their detection have been introduced [16], [30]–[35].

In Appendix S1 other statistical properties of the communities are investigated.

## Discussion

Since the advent of the science of complex networks, its focus has shifted from understanding the emergence and importance of system-level characteristics to mesoscopic properties of networks. These are manifested in communities, *i.e.* densely connected subgraphs. Communities are ubiquitous in networks and typically play an important role in the function of a complex system – modules in protein-interaction networks relate to specific biological functions, and communities in social networks represent the fundamental level of organization in a society. The dual problem of formally defining and accurately detecting communities has so far attracted the most of attention, at the cost of a lack of understanding of the fundamental structural properties of communities. Our aim in this paper has been to uncover some of these properties.

Our results indicate that communities detected in networks of the same class display surprisingly similar structural characteristics. This is remarkable, as some classes are really broad and comprise systems of different origin (e.g. the class of information networks, which includes graphs of citation, co-purchasing and the Web). The result is verified by two different community detection methods which are both partition-based but rely on entirely different principles. In accordance with earlier results, community size distributions are broad for all systems we have studied. Link densities within communities depend strongly on the network class. The average shortest path length displays similar behavior across all classes, initially increasing logarithmically as a function of community size (microcommunities) and then slowing down or saturating for communities of size 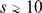 (macrocommunities). In combination with our results on link density in communities, the behavior of path lengths reveals a picture where high-degree nodes are very dominant in communities of certain classes (communication, Internet) and play a less important role in the connectivity of others, especially social networks. This picture is corroborated by the analysis of maximal community-internal degrees of nodes. Finally, also the probability distribution of the fraction of internal links for nodes displays a clear signature for each of the considered classes.

The signatures we have found are a sort of network ID, and could be used both to classify other systems and to identify new network classes. Moreover, they could become essential elements of network models, with the advantage of more accurate descriptions of real networks and predictions of their evolution.

Although our results have been obtained using two different methods, their general validity merits some discussion. As the concept of “community” is ill-defined, every method for detecting communities is based on a specific interpretation of the concept. Furthermore, the underlying philosophies of methods can largely differ. Methods requiring that communities are “locally” very dense, such as clique percolation [16], would detect only a few communities in the communication and Internet networks, as they do not consider trees or stars as communities – nevertheless, this result would be consistent for networks of the same class. On the other hand, it is evident that partition-based methods neglect the fact that nodes may participate in multiple communities. However, it is worth noting that whichever method is used, the resulting communities are actual subgraphs of the network under study, *i.e.* its building blocks. Thus their statistical properties reflect the mesoscopic organization of networks, and our results indicate that this organization is similar within classes of networks.

A very recent paper [36] has arrived to a similar conclusion with an entirely different approach, where taxonomies of networks are constructed based on signatures derived from the modularity of Newman and Girvan.

## Supporting Information

Appendix S1 Appendix to the manuscript.
(0.40 MB PDF)
