# Supplementary material for: Characterizing the Community Structure of Complex Networks
Source: PLoS One. 2010 Aug 12;5(8):e11976. doi: 10.1371/journal.pone.0011976 (PMC2920821; doi:10.1371/journal.pone.0011976)
Supplement: Appendix S1 — Appendix to the manuscript. (0.40 MB PDF) [file pone.0011976.s001.pdf]

Appendix S1: Characterizing the community structure of complex networks

This Appendix is divided in six sections. In Sec. 1, more details regarding some basic statistical properties of the data sets are provided. In Sec. 2, we give a more detailed description of the community detection algorithms. In Sec. 3, we present the same distributions and curves as in the main paper, obtained using the Label Propagation Method (LPM). In Sec. 4 and Sec. 5, we show a number of additional measures regarding statistical properties of modules and nodes. Tables with the fitted exponents for the community size distributions are given in Sec. 6.

1 Data sets: basic statistics

In Fig. S1 we show the degree distributions for all the networks. The degree distribution spans several orders of magnitude. In table S1 we give the exponents of power-law fits carried out with maximum likelihood estimators [1]. In Fig. S2 the clustering coefficient [2] of nodes with degree k is plotted as a function of k, defined as the number of links between neighbors t of the node divided by the maximum possible number of such links f: $c = t/(\frac{1}{2}k(k-1))$. As we can see, the shape of the clustering spectrum is basically the same across all networks, with a rapid decrease of the clustering coefficient with k, except for the Web graphs, which are known to include very dense subgraphs and cliques, for which the clustering coefficient can be appreciably high also for nodes of degree ~ 100. In Fig. S3 we report the average degree k_{nn} of the neighbors of nodes with degree k again as a function of k [3]. Communication networks, the Internet and the Web graphs are clearly disassortative, the other networks are either moderately disassortative or do not exhibit a particular correlation. Only the Livejournal friendship network has an assortative pattern for intermediate degree-values.

Network degree distribution					
Category	name	exponent	min degree	exp error	p—value
Communication	wikitalk	-2.46	1	0.01	0
	email	-2.93	1	0.01	0
Internet	caida	-2.12	5	0.03	0.6
	dimes	-2.2	2	0.01	0.2
Information	Web Google	-2.68	23	0.01	0.8
	arxiv	-3.19	61	0.04	0.3
	amazon	-3.27	17	0.03	0
	Web BS	-2.59	46	0.02	0
Biological	dmela	- 3.50	28	0.05	0
	yeast	-3.0	5	0.5	0.4
	human	3.0	31	0.2	0.1
Social	live j.	-2.8	86	0.1	0.3
	epinions	-1.70	1	0.01	0
	last fm	-2.9	35	0.1	0
	slashdot	-2.5	43	0.5	0.8

Table S1: List of the networks used for our analysis together with the exponent of the degree distribution and the minimum degree from which the fit holds. We used maximum likelihood fitting [1].

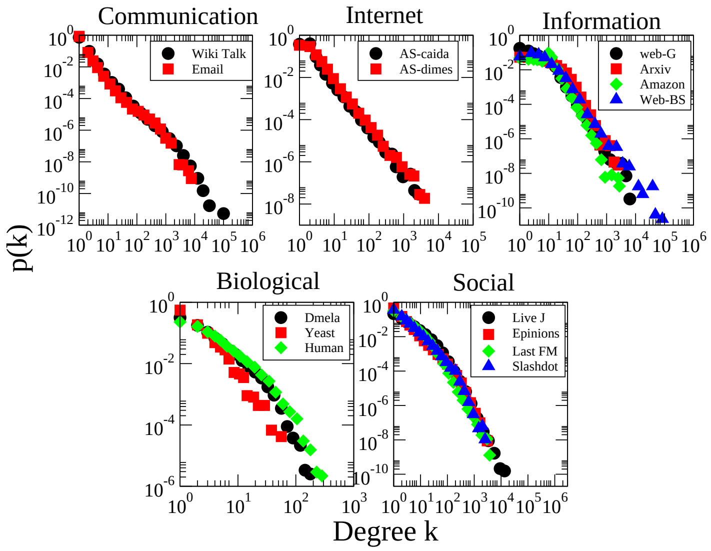

Figure S1: Degree distributions.

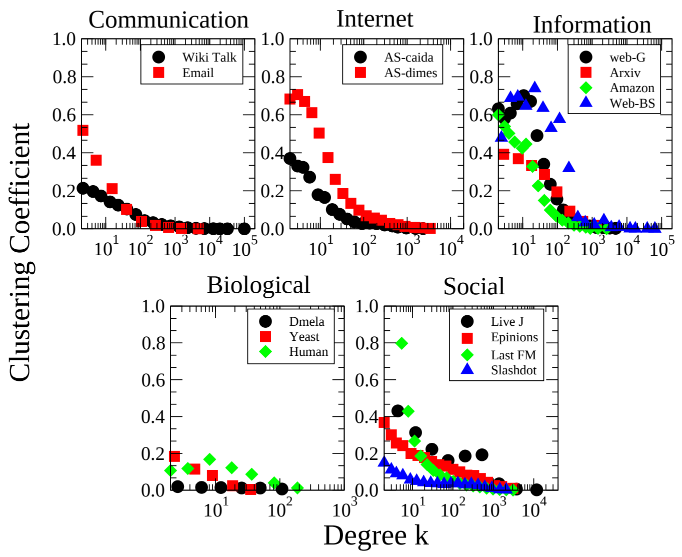

Figure S2: Clustering coefficient versus degree.

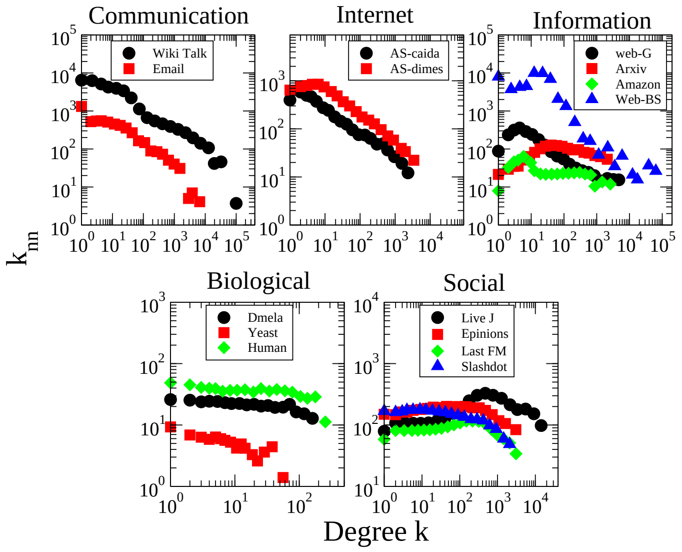

Figure S3: Average nearest-neighbor degree k_{nn} versus degree.

2 The community detection methods

In this section, we briefly explain the two community detection algorithms. For a detailed description, the reader is referred to the original publications.

Infomap [4] is based on the idea that a random walker exploring the network should get trapped inside dense modules for a fairly long time, and cross the boundaries of modules only infrequently. This simple idea is formalized by considering the problem of finding the optimal description of the path of the walker, which can be achieved by labelling every node with a prefix given by a unique name for the module it belongs to and a suffix given by a unique name within its module. The labels of nodes, while unique within their module, can be recycled in different modules to achieve the most compressed description. According to such two-level description, given a partition of the graph, one can compute the amount of information needed to describe the path of the walker. If the network has a well-defined community structure, the code length of the two-level description may be shorter than the code length of the one-level description, in which each node has a unique name, as the walker will perform most of its steps within each module and comparatively few between the modules. In this way, the recycling of the labels leads to a more compact description of the process. Then the problem of Infomap is finding the partition which gives the smallest description length. This optimization problem is solved using a greedy optimization algorithm in order to obtain the results in reasonable time. The use of random walks makes the method naturally generalizable to the case of directed and weighted graphs. For directed graphs, due to the possibility of having dangling ends, which are sinks for the diffusion process, it is necessary to introduce a teleportation factor, similarly to Google's PageRank algorithm [5].

The Label Propagation Method [6] basically simulates the spreading of labels based on the simple rule that at each iteration a given node takes the most frequent label in its neighborhood. The starting configuration is chosen such that every node is given a different label and the procedure is iterated until convergence. This method has the problem of partitioning the network such that there are very big clusters, due to the possibility of a few labels to propagate over large portions of the graph. The LPM version that we used in our analysis is a modification by Leung et al. [7] that handles this problem by introducing a hop score which tells how far a certain label is from its origin. The hop score is decreased while the label spreads through the network and this improves the quality of the partitions found by the method.

3 Main results from the Label Propagation Method

In order to verify that our results are not due to the method alone, but represent real features of the mesoscopic organization of the networks, we have carried the analysis presented for Infomap in the main paper with the Label Propagation method as well. The following plots show the characteristics presented in the main paper obtained via the label propagation method. Results are consistent with those obtained with Infomap.

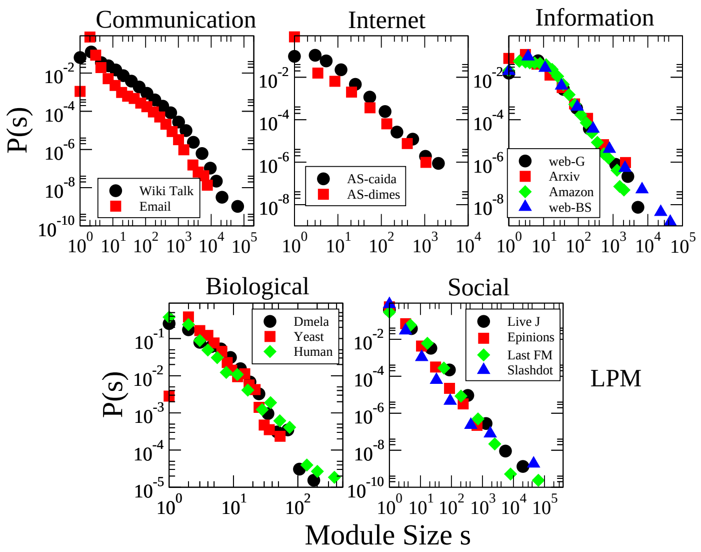

Figure S4: Distribution of community sizes.

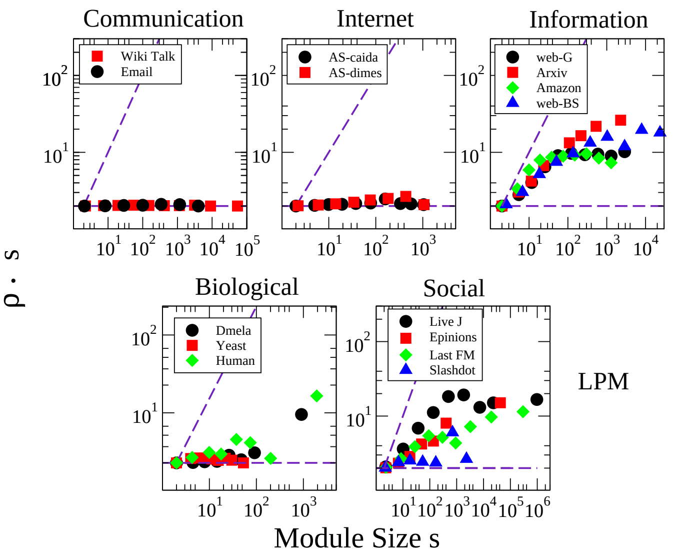

Figure S5: Scaled link density of communities as a function of the community size.

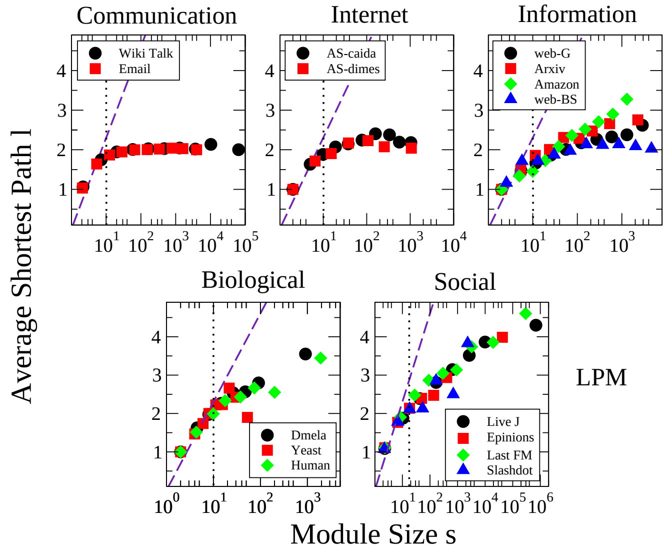

Figure S6: Average shortest path of a community as a function of the community size s.

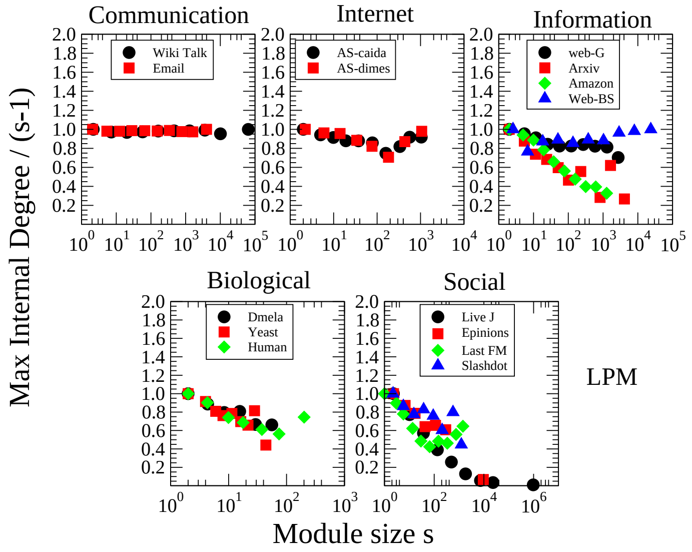

Figure S7: Ratio between the maximum internal degree $\max(k_{in})$ of a node and the maximum possible number of internal neighbors $s-1$ as a function of s, the module size.

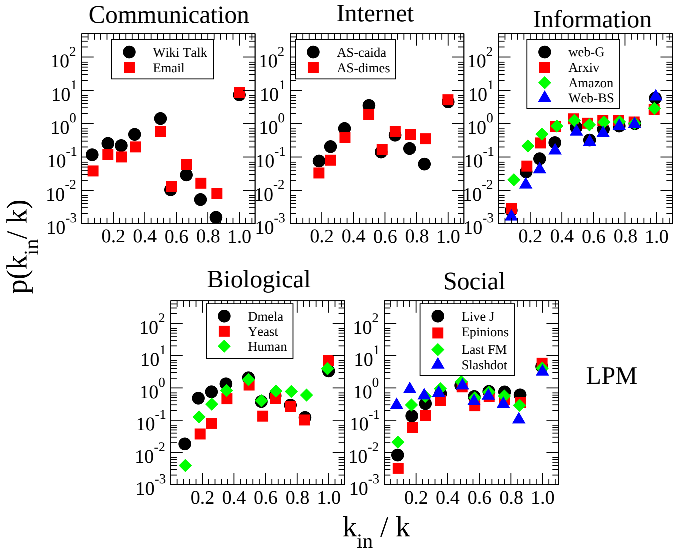

Figure S8: Distribution of the fraction of neighbors of a node belonging to the community of the node.

4 Further Statistics on Community Properties

In this section, we want to show some other statistical properties of the modules. All figures display the results obtained using Infomap (upper panel) and the Label Propagation Method (lower panel).

As in the main article only the average values of link densities are shown, we first want to show what the probability distribution of the link density ρ of communities looks like. Fig. S9 shows that in all the systems there are dense modules together with sparser modules. Nevertheless, there is a dependency on the size of the modules: Fig. S10 shows what happens if we discard very small communities, with less than 3 nodes ($s < 3$), and Fig. S11 displays what is left when we consider fairly big modules, $s > 10$; only social and information networks include dense modules even after this filtering.

Next, we show the average internal clustering coefficient as a function of the module size s, Fig. S12. The clustering coefficient c is a node property defined as the number of links between neighbors t of the node divided by the maximum possible number of such links for a node with the same degree k: $c = t/(\frac{1}{2}k(k-1))$. For nodes with degree smaller than two we consider the clustering coefficient to be undefined and leave them out of the calculations of the averages. Here, "internal" means that the clustering coefficient is computed by only considering the subgraph of the community, which includes only the internal links in the community. For communication systems and the Internet, the average internal clustering coefficient of large communities can reach fairly high values although the corresponding densities ρ are low. This can be explained in terms of "merged-star" structures, where two (or more) high-degree nodes are connected, their neighbours have a low degree (approx. the number of hubs) and are connected to all hubs. As then the clustering coefficient for these nodes is typically unity and their number is large, they dominate the average clustering coefficient within the community.

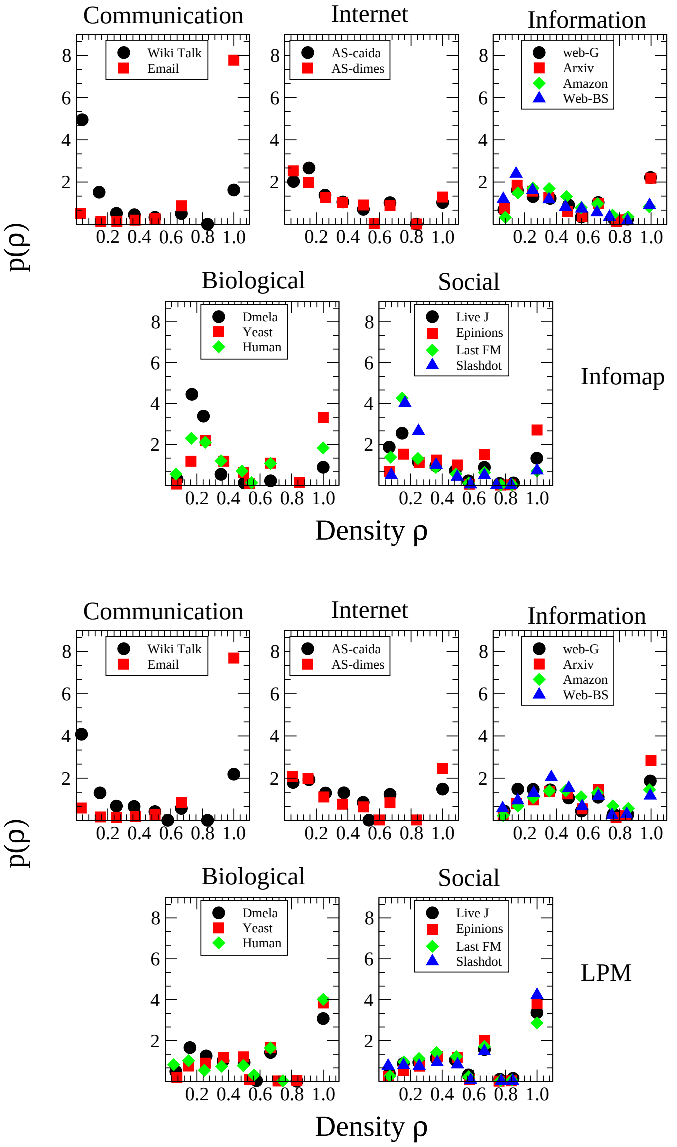

Figure S9: Distribution of the link density for $s > 1$.

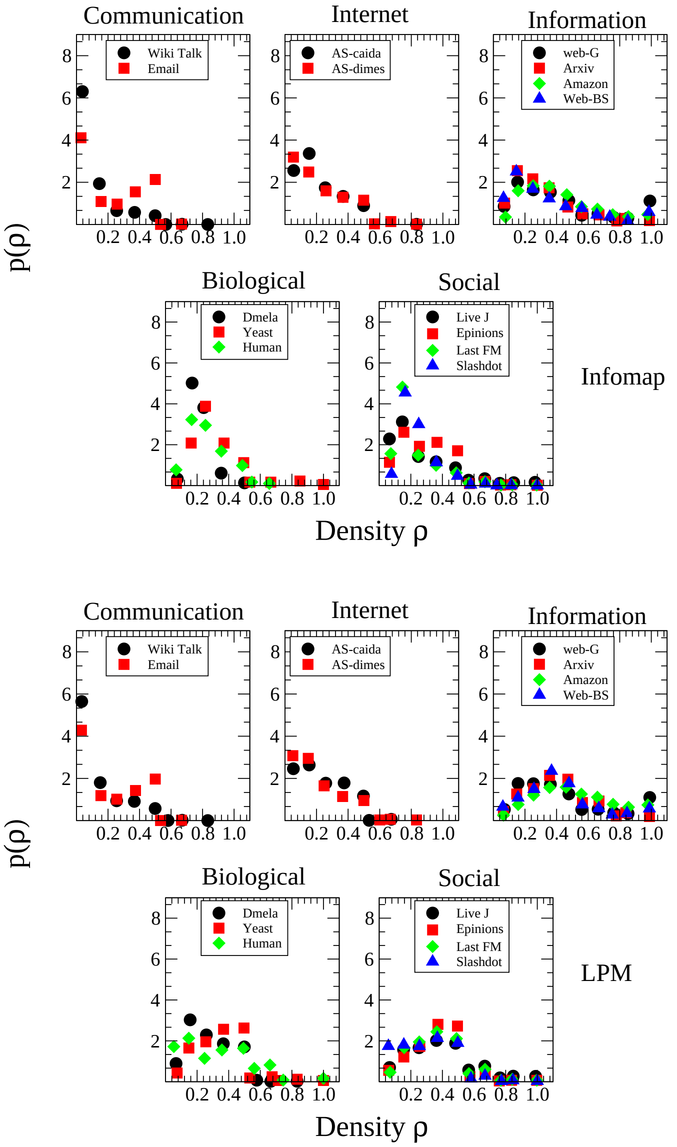

Figure S10: Distribution of the link density for s > 3.

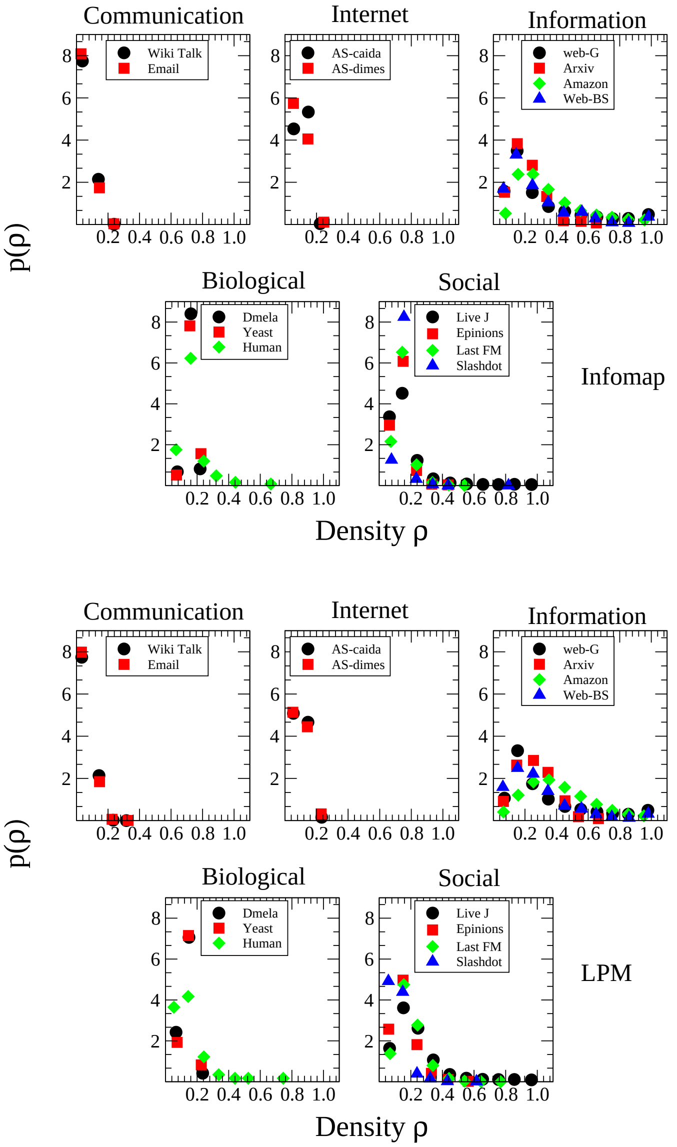

Figure S11: Distribution of the link density for s > 10.

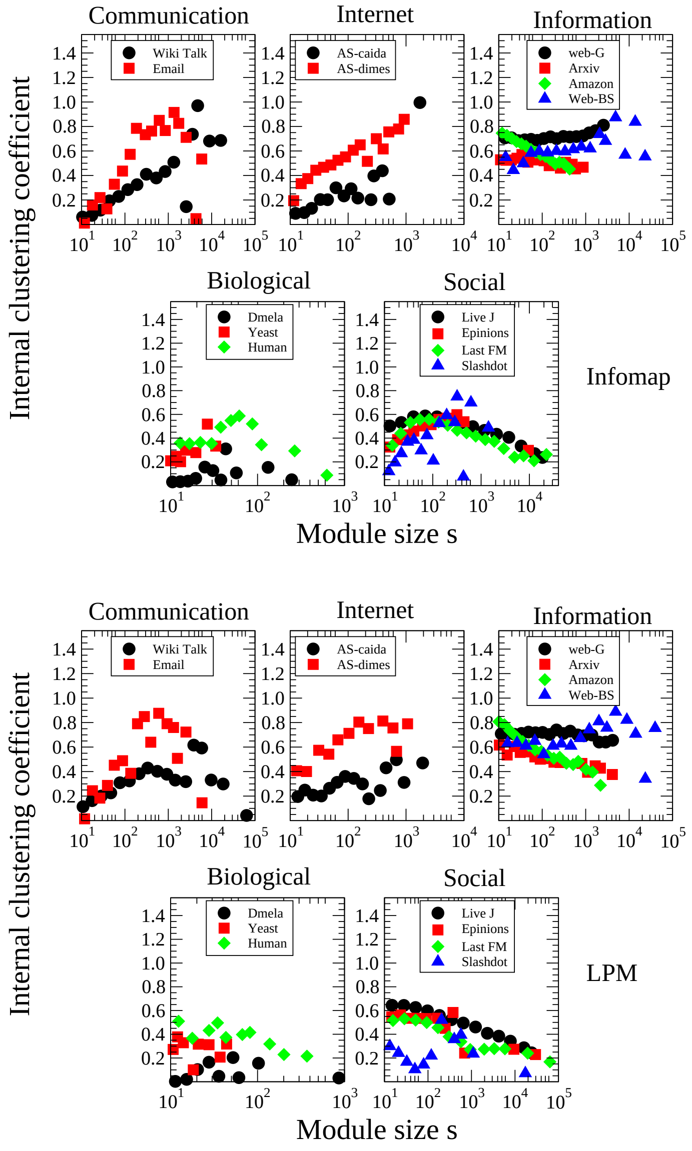

Figure S12: Internal clustering coefficient as a function of the module size.

5 Further Statistics of Node Properties

Here we focus on properties of nodes with respect to their communities. Again, we show results obtained using both Infomap (upper panel) and the Label Propagation Method (lower panel).

In Fig. S13, we show the distribution of the fraction of neighbors of a node belonging to its community when one only considers nodes with degree $k > 3$ (in the main manuscript we considered all nodes). Fig. S14 is the same plot, but including only nodes with degrees larger than 10. The two plots display flatter curves than the full distributions, and they look much smoother, indicating that the fluctuations observed in the full curves are mostly due to low degree nodes. Low degree nodes can cause peaks in the plots because the values of the fraction k_{in}/k are quantized (*e.g.* for a node of degree two the fraction must be 0, 0.5 or 1). By observing the rightmost points of the curves, we see that they lie much lower than the corresponding points of the full curves, except for the information networks. This means that many nodes which are fully embedded in their community have low degree, as expected.

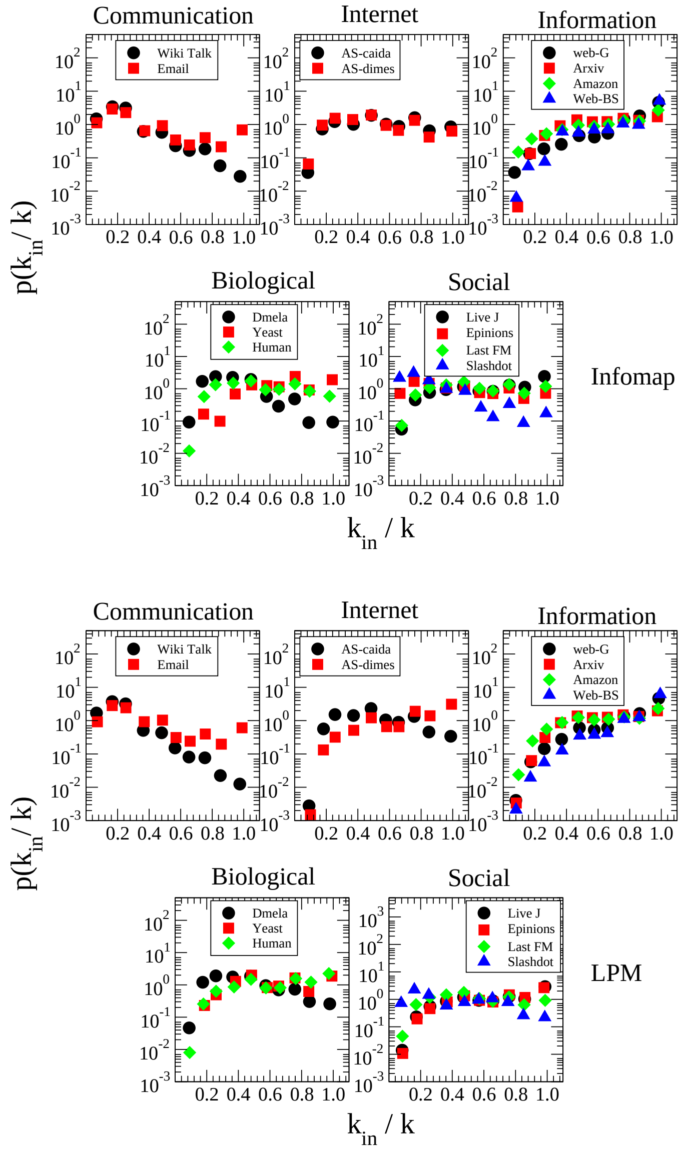

Figure S13: Distribution of k_{in}/k, for $k > 3$.

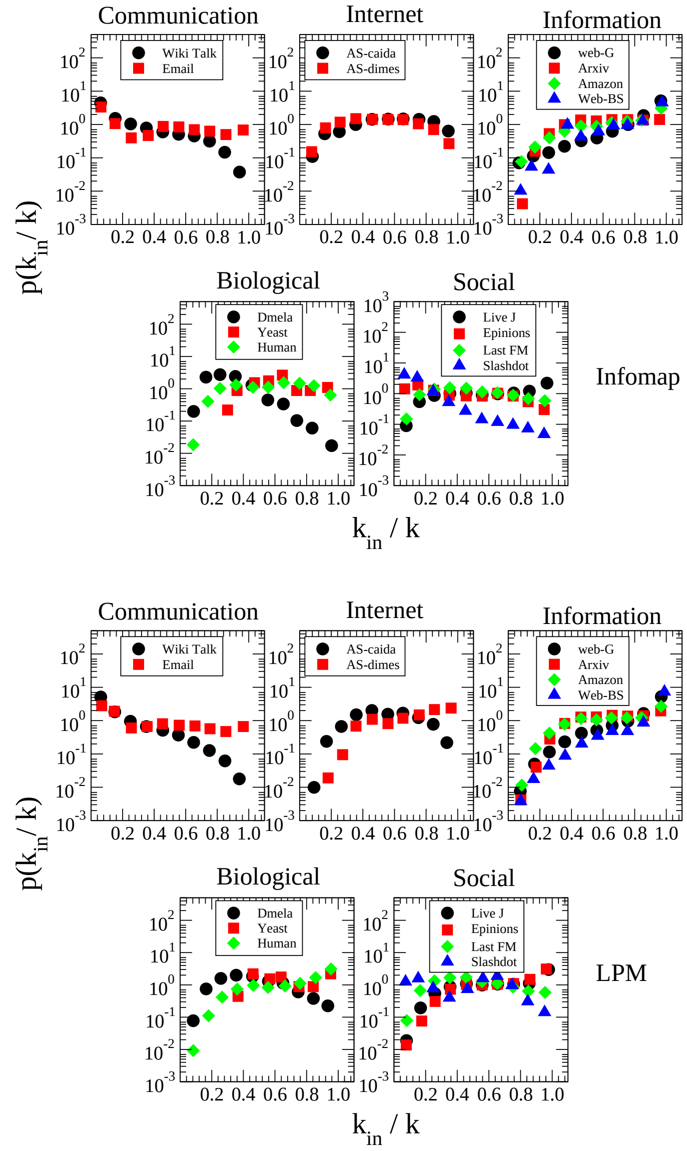

Figure S14: Distribution of k_{in}/k, for $k > 10$.

6 Fitted exponents for the community size distribution

In the following tables, we collected the power-law exponents which best fit the tail of the community size distribution, if the tail is assumed to be of the form $p(s) \propto s^{-\alpha}$. These were obtained using the maximum likelihood method. p-values are also shown, indicating that in many cases it is unlikely that the distributions are power laws.

Community size distribution (from Infomap)					
Category	name	exponent	min degree	exp error	p−value
Communication	wikitalk	-2.7	881	0.3	0.1
	email	-2.8	674	0.3	0.5
Internet	caida	-2.10	11	0.05	0.4
	dimes	-2.00	18	0.05	0.9
Information	Web Google	-2.57	89	0.03	0.2
	arxiv	-2.4	69	0.3	0.5
	amazon	-3.5	97	0.2	0.02
	Web BS	-2.4	36	0.1	0
Biological	dmela	- 3.5	9	0.1	0.1
	yeast	-3.05	8	0.05	0.1
	human	2.6	8	0.1	0.1
Social	live j.	-2.22	59	0.02	0
	epinions	-2.5	13	0.2	0.3
	last fm	-2.70	34	0.05	0
	slashdot	-3.5	10	0.1	0

Community size distribution (from LPM)					
Category	name	exponent	min degree	exp error	p−value
Communication	wikitalk	-2.6	1145	0.2	0.4
	email	-2.4	248	0.1	0.2
Internet	caida	-2.08	13	0.08	0.4
	dimes	-1.95	12	0.05	0.8
Information	Web Google	-2.45	36	0.02	0.1
	arxiv	-2.0	16	0.1	0.1
	amazon	-2.80	30	0.05	0.3
	Web BS	-2.0	107	0.1	0.7
Biological	dmela	- 2.7	10	0.5	0.3
	yeast	-2.6	5	0.5	0.3
	human	1.9	2	0.05	0.2
Social	live j.	-2.40	86	0.05	0.1
	epinions	-2.40	5	0.05	0.2
	last fm	-2.9	35	0.1	0.1
	slashdot	-2.7	24	0.1	0

References

[1] Clauset A, Shalizi CR, Newman MEJ (2009) Power-law distributions in empirical data. SIAM Review 51: 661-703.

[2] Watts DJ, Strogatz SH (1998) Collective dynamics of 'small-world' networks. Nature 393: 440-442.

[3] Pastor-Satorras R, Vazquez A, Vespignani A (2001) Dynamical and correlation properties of the Internet. Phys. Rev. Lett. 87: 258701.

[4] Rosvall M, Bergstrom CT (2008) Maps of random walks on complex networks reveal community structure. Proc. Natl. Acad. Sci. U.S.A. 105: 1118-1123.

[5] Brin S, Page L (1998) The anatomy of a large-scale hypertextual Web search engine. Comp. Netw. 30: 107-117.

[6] Raghavan UN, Albert R, Kumara S (2007) Near linear time algorithm to detect community structures in large-scale networks. Phys. Rev. E 76: 036106.

[7] Leung IXY, Hui P, Lió P, Crowcroft J (2009) Towards real-time community detection in large networks. Phys. Rev. E 79: 066107.
